# Serum progranulin is not associated with rs5848 polymorphism in Korean patients with neurodegenerative diseases

**DOI:** 10.1371/journal.pone.0261007

**Published:** 2022-01-27

**Authors:** Na-Yeon Jung, Hyang-Sook Kim, Eun Soo Kim, Sumin Jeon, Myung Jun Lee, Kyoungjune Pak, Jae-Hyeok Lee, Young Min Lee, Kangyoon Lee, Jin-Hong Shin, Jun Kyeung Ko, Jae Meen Lee, Jin A. Yoon, Chungsu Hwang, Kyung-Un Choi, Gi Yeong Huh, Young-Eun Kim, Eun-Joo Kim

**Affiliations:** 1 Department of Neurology, Pusan National University Yangsan Hospital, Pusan National University School of Medicine, Yangsan, Republic of Korea; 2 Research Institute for Convergence of Biomedical Science and Technology, Pusan National University Yangsan Hospital, Yangsan, Republic of Korea; 3 Department of Anesthesia and Pain Medicine, School of Medicine, Pusan National University, Busan, Republic of Korea; 4 Department of Neurology, Pusan National University Hospital, Pusan National University School of Medicine and Medical Research Institute, Busan, Republic of Korea; 5 Department of Nuclear Medicine, Pusan National University Hospital, Pusan National University School of Medicine, Busan, Republic of Korea; 6 Department of Psychiatry, Pusan National University Hospital, Pusan National University School of Medicine, Busan, Republic of Korea; 7 Department of Neurosurgery, Medical Research Institute, Pusan National University Hospital, Busan, Republic of Korea; 8 Department of Rehabilitation Medicine, Pusan National University School of Medicine and Biomedical Research Institute, Pusan National University Hospital, Busan, Republic of Korea; 9 Department of Pathology, Pusan National University School of Medicine, Yangsan, Republic of Korea; 10 Department of Forensic Medicine, Pusan National University School of Medicine, Yangsan, Republic of Korea; 11 Department of Laboratory Medicine, Hanyang University College of Medicine, Seoul, Republic of Korea; Sorbonne Universite, FRANCE

## Abstract

Low serum progranulin (PGRN) is known to be associated with granulin (*GRN*) gene mutation and T alleles of *GRN* rs5848 polymorphism. However, there have been only a few Asian studies exploring these. We investigated the serum PGRN levels, rs5848 genotypes, and their relations with cerebrospinal fluid (CSF) Alzheimer’s disease (AD) biomarkers in the Korean population. Serum PGRN levels, *GRN* rs5848 polymorphism, and *GRN* mutations were evaluated in 239 participants (22 cognitively unimpaired participants and 217 patients with neurodegenerative diseases). CSF AD biomarkers were also evaluated in 214 participants. There was no significant difference in the serum PGRN levels among the diagnostic groups. We could not find any *GRN* mutation carrier in our sample. The differences in the frequencies of the rs5848 genotypes among the clinical groups or the effects of the rs5848 genotypes on serum PGRN were not observed. There was no correlation between the serum PGRN level or rs5848 genotype and CSF AD biomarkers. Neither the T allele nor the TT genotype had an effect on the development of AD. Our results showed that serum PGRN levels were not associated with rs5848 genotypes, indicating that multiple single nucleotide polymorphisms might affect PGRN concentrations in an ethnicity-specific manner.

## Introduction

Progranulin (PGRN) is widely expressed in many tissues, including the neuron and microglia of the central nervous system. Although the exact function of PGRN in the brain remains unclear, previous studies suggested that PGRN might serve as a neurotrophic factor [1] and the down-regulation of PGRN might lead to neurodegeneration [2, 3]. The role of PGRN in the pathogenesis of Alzheimer’s disease (AD) remains unclear. In several animal studies, low PGRN levels have been associated with increased tau pathology. However, the effects of PGRN on Aβ pathology are inconsistent [4–6].

The PGRN gene (*GRN*) mutation is one of the major genetic causes of frontotemporal lobar degeneration-TAR DNA-binding protein (FTLD-TDP) [7]. The pathogenic mechanism of *GRN* mutation is the loss of 50% functional PGRN [3, 8]. Based on this haplo-insufficiency mechanism, several studies reported reduced PGRN levels in the cerebrospinal fluid (CSF) or blood of patients with *GRN* mutations and proposed PGRN level as a useful biomarker for predicting *GRN* mutations [1, 9–11].

The rs5848, a common *GRN* variant, was associated with an increased risk of AD, frontotemporal dementia (FTD), and Parkinson’s disease (PD) [12–15]. The T allele of rs5848 increases the binding of miR-659 to PGRN mRNA, leading to translational repression and reduced PGRN expression [15–17]. Although previous meta-analyses showed significant associations between rs5848 and an increased risk of neurodegenerative diseases [14, 18], only a few studies have investigated the role of rs5848 in neurodegenerative diseases in Asian populations [12, 13].

In this study, we firstly examined whether serum PGRN level differed across clinical syndromes and whether these levels could be used to predict GRN mutations in the Korean population. Given that pathogenic variants of *GRN* are rare in Korean patients with FTD [19, 20] and mutations of *GRN* are associated with highly variable clinical phenotypes including amnestic syndromes (AD dementia, mild cognitive impairment (MCI), hippocampal sclerosis), corticobasal syndrome, and amyotrophic lateral sclerosis, as well as FTD syndromes [21, 22], we hypothesized that screening tests for serum PGRN in various neurodegenerative dementia would be relevant for identifying Korean *GRN* mutation carriers who possibly presented with various neurodegenerative dementia other than FTD syndromes. Secondly, to better understand the relationship between PGRN and AD pathology, we evaluated whether serum PGRN level was related to CSF AD biomarkers. Lastly, we explored whether the T allele of rs5848 was associated with low levels of serum PGRN levels and increased risk of AD.

## Materials and methods

### Participants

This study consecutively recruited 239 participants who attended the Dementia Clinics at two tertiary referral hospitals (Pusan National University Hospital and Pusan National University Yangsan Hospital) between April 2015 and January 2019. Examination of all participants by neurologists who specialized in neurodegenerative diseases was followed by clinical interview and neurological examination. Cognitive assessment included the Mini-Mental State Examination (MMSE) and Seoul Neuropsychological Screening Battery (SNSB) [23]. The participating 239 patients consisted of 74 patients with AD dementia (ADD) [24], 47 with MCI [25, 26], 30 with normal pressure hydrocephalus (NPH) [27], 16 with FTD [28, 29], 7 with PD [30], 16 with subjective memory impairment (SMI) [31], 27 with other neurodegenerative diseases, and 22 cognitively unimpaired (CU) participants with no history of neurological or psychiatric diseases. The 27 patients with other neurodegenerative diseases included two patients with progressive supranuclear palsy-Richardson syndrome (PSP-RS) [32], nine with dementia with Lewy bodies (DLB) [33], one with progressive bulbar palsy, one with epileptic memory impairment, three with spinocerebellar ataxia, two with vascular cognitive impairment, five with unclassified neurodegenerative dementia, and four with unclassified parkinsonism. Two patients with vascular cognitive impairment showed moderate to severe white matter hyperintensities without any space occupying lesion such as infarction, hemorrhage or tumor on MRI. Three patients with spinocerebellar ataxia showed cognitive impairment and motor symptoms (e.g., cerebellar ataxia). Complete blood count, biochemistry, thyroid function tests, lipid profiles, vitamin B12 and folate levels, and tests for syphilis were performed for all participants to exclude metabolic causes of cognitive impairment. To exclude territorial cerebral infarction, brain tumor, and other structural lesions, either magnetic resonance imaging or computed tomography of the brain was conducted for all participants.

This study was approved by the institutional review boards of Pusan National University Hospital and Pusan National University Yangsan Hospital. Written informed consent was obtained from each participant or their caregiver prior to enrollment in the study.

### Assessment of serum PGRN

Blood samples from each participant were collected in plain and EDTA tubes, and serum and buffy coat were separated via centrifugation (2000 *×g*, 10 min, 4°C). The serum was divided into 250 μL aliquots and frozen at -80°C. Assays were performed on the sample aliquots after a single thawing process. Serum PGRN levels were measured using a commercial ELISA kit (Human Progranulin ELISA kit, AdipoGen, Inc., South Korea) according to the manufacturer’s instructions. The experiments were conducted in duplicate and were blinded to the clinical diagnosis. Serum samples were diluted 1:200 prior to analysis.

### Sanger sequencing and genotyping

The buffy coat, which was separated, stored in aliquots (1 mL), and frozen at -80°C, was used for DNA sequencing. Genomic DNA was extracted from the peripheral blood leukocytes of participants using the Wizard Genomic DNA Purification Kit (Promega, A1120, Madison, USA). We performed Sanger sequencing for *GRN* all coding exons and flanking region of *GRN* gene, and genotyping of single nucleotide polymorphism (SNP) rs5848. The PCR primers used were: *GRN* exon 2–4 (forward primer: 5′-TGAGTGACCCTAGAATCAAGG-3′; reverse primer: 5′-ACATGAATGAGGGCACAAGG-3′); *GRN* exon 5–7 (forward primer: 5′-GAGTCACCTTCCCTGAGTG-3′; reverse primer: 5′-CTGTAAGGTGCGTGTCAGG-3′); *GRN* exon 8–10 (forward primer: 5′-TGATGCAGGGTTCATGCTAC-3′; reverse primer: 5′-GTATCACCTATGGGCTATGC-3′); *GRN* exon 11–13 (forward primer: 5′-AGGTGCTGTAAGCAGGAGAG-3′; reverse primer: 5′-GGATAGGGAAAAGCACCTGG-3′); and *GRN* rs5848 (forward primer: 5′-TTTGAGGGACCCAGCCTTG-3′; reverse primer: 5′-GGATAGGGAAAAGCACCTGG-3′). The amplification products were sequenced on an Applied Biosystems 3730xl DNA Analyzer using the Big Dye Terminator v3.1 Cycle Sequencing kit (Applied Biosystems, Foster, CA, USA). The PCR amplification parameters used were: 95°C for 2 min and 35 cycles at 95°C for 30 s, 58°C for 30 s, and 72°C for 1 min.

*APOE* genotyping was performed with real-time quantitative PCR using either a Real-Q Apo E genotyping kit (BioSewoom, Seoul, Korea) or a DiaPlexC^™^ Apolipoprotein E Genotyping Kit (SolGent, Daejeon, Korea) according to the manufacturer’s recommendations.

### CSF AD biomarker assessment

Lumbar punctures were performed to collect CSF samples from 214 participants. Of these, 179 participants in our previous study [34]. The 214 participants were composed of 27 CU participants, 42 patients with MCI, 71 with ADD, 15 with FTD, 6 with PD, 23 with other neurodegenerative diseases, and 30 with NPH. The CSF samples were analyzed at the Research Institute for Convergence of Biomedical Science and Technology at PNUYH. CSF beta amyloid 1–42 (Aβ_1–42_), total tau (t-tau), and phosphorylated tau (p-tau) levels were quantified using the INNOTEST ELISA kit (Fujirebio Diagnostics, Ghent, Belgium). Detailed methods and cut-off values for each biomarker for the diagnosis of ADD have been described elsewhere [34]. Based on the ATN classification suggested by the National Institute on Aging and Alzheimer’s Association Research Framework [35], each participant was classified into three binary categories. A+ refers to Aβ pathology (CSF Aβ_1–42_ < 631.8 pg/mL), T+ refers to phosphorylated tau pathology (CSF p-tau> 56.4 pg/mL), and N+ refers to neurodegeneration (CSF t-tau > 271.6 pg/mL) [34].

### Statistical analysis

One-way analysis of variance (one-way ANOVA) with Bonferroni post-hoc correction for multiple comparisons was used to analyze the differences in continuous variables (age, education, MMSE scores, and serum PGRN levels) among the clinical diagnostic groups. To analyze the differences in categorical variables (sex, *APOE*ε4, and rs5848 genotype), we used the chi-squared test or Fisher’s exact test. The relationships between the demographic factors (age, sex, MMSE scores, and *APOE*ε4) and serum PGRN levels were explored by multiple linear regression analysis.

Serum PGRN levels among the different rs5848 genotypes were compared by ANCOVA after controlling for age, sex, and *APOE*ε4 genotype. The CSF AD biomarkers among the rs5848 genotypes were compared by ANCOVA after controlling for potential confounders (age, sex, diagnosis, and *APOE*ε4 genotype). CSF biomarkers were log-transformed because they were not normally distributed. To examine the association between serum PGRN and AD biomarkers, we performed Spearman’s rank correlation analysis. The risk estimate for developing AD in participants with the T allele was calculated using binary logistic regression. All statistical analyses were conducted using the Statistical Package for the Social Sciences 25 (SPSS Inc.).

## Results

### Demographic and laboratory characteristics

The demographic characteristics of the clinical diagnostic groups are presented in Table 1. Participants in the NPH group were significantly older than those in the SMI, MCI, ADD, FTD, and PD groups. The FTD and other neurodegenerative disease groups had more male participants than the CU, MCI, and PD groups. The ADD group had a lower mean MMSE score than the CU, SMI, MCI, and PD groups. There was no significant difference in the frequency of the *APOE*ε4 allele across the diagnostic groups.

**Table 1:** Demographic characteristics and serum PGRN level according to clinical diagnostic groups.

	CU	SMI	MCI	ADD	FTD	PD	Others	NPH	p value
N	22	16	47	74	16	7	27	30	
Age	68.1 (7.7)	63.8 (6.5)	67.9 (11.5)	68.5 (9.7)	65.0 (11.2)	63.0 (9.6)	67.9 (9.3)	75.6 (4.8)†	0.001
Gender (female, %)	16 (72.7)	11 (68.8)	31 (66.0)	42 (56.8)	3 (18.8)‡	7 (100)	7 (25.9)*	14 (46.7)	<0.001
MMSE	28.0 (2.3)	26.2 (3.2)	25.0 (2.9)	16.5 (6.9)^a^	18.8 (8.4)^b^	25.0 (5.8)	20.4 (6.9)^c^	18.9 (5.7)^d^	<0.001
APOE ε4 carrier (%), n = 169	3 (23.1)	6 (42.9)	17 (37.8)	23 (39.7)	2 (16.7)	0 (0)	6 (37.5)	1(10)	0.446
Serum PGRN levels (ng/mL)	112 (25)	134 (28)	134 (37)	128 (36)	137(43)	134 (29)	125 (31)	134 (33)	0.279

*P-values* were calculated using ANOVA, with Bonferroni post-hoc correction or Fisher’s exact test (or chi-squared test).

Data are expressed as the mean (standard deviation) or number of subjects (%).

^†^The NPH group was older than the SMI, MCI, ADD, FTD, and PD groups.

^‡^The FTD group had more male participants than the CU, MCI, and PD groups.

*The others group had more male participants than the CU, MCI, and PD groups.

^a^The ADD group had lower MMSE scores than the CU, SMI, MCI, and PD groups.

^b^The FTD group had lower MMSE scores than the CU, SMI, and MCI groups.

^c^The others group had lower MMSE scores than the CU, SMI, and MCI groups.

^d^The NPH group had lower MMSE scores than the CU, SMI, and MCI groups.

Abbreviations: ADD, Alzheimer’s disease dementia; APOE, apolipoprotein E; CU, cognitively unimpaired people; FTD, frontotemporal dementia; MCI, mild cognitive impairment; MMSE, mini mental state examination; NPH, normal pressure hydrocephalus; PD, Parkinson’s disease; PGRN, progranulin; SMI, subjective memory impairment.

### Analysis of serum PGRN levels and the *GRN* mutation

The mean PGRN level for all participants was 129.3 ng/mL (65.2–282.5 ng/mL). The mean PGRN level in the CU group was 112.3 ng/mL (68.9–152.5 ng/mL). There was no significant difference in the serum PGRN levels among the clinical diagnostic groups (Table 1). The effects of age (B = 0.13, p = 0.644), sex (B = 5.927, p = 0.278), MMSE scores (B = 0.426, p = 0.27), and *APOE*ε4 genotype (B = 7.905, p = 0.166) on the serum PGRN levels were not observed in multiple linear regression analysis. Direct sequencing of *GRN* did not reveal any novel pathogenic variant but led to the identification of five heterozygous missense variants and one in-frame deletion of uncertain significance (VUS) in six patients (S1 Table and S1 Fig): exon 6 c.530G >A (p.Arg177His) in two patients with NPH (V2 and V3); exon 7 c.662G >C (p.Cys221Ser) in a patient with ADD (V4); exon 8 c.827C >T (p.Ala276Val) in a patient with unspecified dementia (V5), exon 13 c.1690C >T (p.Arg564Cys) in a CU participant (V6), and exon 5 c.355_357del (p.Asn119del) in a patient with ADD (V1). These variants were not detected by restriction fragment length polymorphism (RFLP) or direct sequencing in 100 control samples. The mean PGRN level in the six patients with VUS was 128.9 ng/mL (93.5 ng/mL–169.0 ng/mL). The patient with the lowest PGRN level (93.5 ng/mL) was clinically diagnosed with ADD and had positive CSF AD biomarkers (A+T+N+) and *APOE* ε3/ε4. The other patient with ADD and positive AD biomarkers (V1) had moderately low PGRN level (118.7 ng/mL). However, the patient with unspecified dementia and negative AD biomarkers (A-T-N-) (V5) also had moderately low PGRN level (101.8 ng/mL).

### Relationship between PGRN and CSF AD biomarkers

Of the 71 participants with ADD who were evaluated for CSF AD biomarkers, 40 had AD pathology (A+T+N+) based on the cut-off values that were previously established in our laboratory [34, 35]. The mean PGRN level for these A+T+N+ patients was 125 ng/mL (SD: 32), which was comparable to that in patients with total ADD (128 ng/mL). We could not find correlations between serum PGRN and CSF Aβ_1–42_, t-tau, and p-tau levels in individual participants or in diagnostic groups (Table 2).

**Table 2:** Association between serum PGRN and CSF biomarkers.

	Aβ_1–42_		T-tau		P-tau	
	rho	p	rho	p	rho	P
Total (n = 214)	0.054	0.433	0.025	0.717	-0.010	0.881
CU (n = 11)	-0.309	0.355	0.591	0.056	0.191	0.574
SMI (n = 16)	-0.162	0.549	-0.347	0.188	-0.153	0.572
MCI (n = 42)	0.264	0.091	0.151	0.34	0.145	0.366
ADD (n = 71)	0.027	0.826	0.083	0.491	0.134	0.267
FTD (n = 15)	0.025	0.930	-0.321	0.243	-0.393	0.164
PD (n = 6)	-0.600	0.208	-0.257	0.623	-0.200	0.747
Others (n = 23)	0.067	0.761	-0.135	0.538	-0.352	0.100
NPH(n = 30)	-0.099	0.602	0.096	0.614	0.164	0.405

Analysis was based on Spearman’s rank correlation.

The relationship between serum PGRN and CSF p-tau levels was evaluated in 208 CU, 41 MCI, 70 ADD, 14 FTD, 5 PD, and 28 NPH, because six patients had undetectable p-tau levels.

Abbreviations: ADD, Alzheimer’s disease dementia; CSF, cerebrospinal fluid; CU, cognitively unimpaired people; FTD, frontotemporal dementia; MCI, mild cognitive impairment; NPH, normal pressure hydrocephalus; PD, Parkinson’s disease; PGRN, progranulin; P-tau, phosphorylated tau; SMI, subjective memory impairment; T-tau, total tau.

### rs5848 genotypes across clinical syndromes

There was no significant difference in the frequencies of the rs5848 genotypes in CU participants, compared with all patients (Fisher’s exact test, p = 0.878), or with different diagnostic groups (chi-squared test, p = 0.656). The most common genotype was the CC genotype (54.5% for the CU participants and 55.3% for the patients), followed by the CT genotype (36.4% for the CU participants and 37.8% for the patients), and the least frequent genotype was the TT genotype (9.1% for the CU participants and 6.9% for the patients).

In the CU, SMI, ADD, PD, NPH, and other neurodegenerative disease groups, the CC genotype was the most common, whereas in the MCI and FTD groups, the CT genotype, followed by the CC and TT genotypes, was the most frequent. The TT genotype was the least frequent in all diagnostic groups (Table 3).

**Table 3:** Comparison of serum PGRN levels within each diagnostic group and each rs5848 genotype.

	N within each genotype			PGRN level in CC (ng/mL)	PGRN level in CT(ng/mL)	PGRN level in TT(ng/mL)	p value
	CC	CT	TT				
Total (n = 239, %)	132 (55.2)	90 (37.7)	17 (7.1)	135±37	121±29	130±32	0.094
CU (n = 22, %)	12 (54.5)	8(36.4)	2(9.1)	116±29	105±19	120±4	0.877
SMI (n = 16, %)	9 (56.3)	6(37.5)	1(6.3)	133±25	138±37	125	0.801*
MCI (n = 47, %)	20(42.6)	22(46.8)	5(10.6)	142±44	123±27	153±38	0.277
ADD (n = 74, %)	47(63.5)	23(31.1)	4 (5.4)	128±36	128±37	126±46	0.769
FTD (n = 16, %)	6(37.5)	7(43.8)	3(18.8)	170±54	116±22	121±14	0.505
PD (n = 7, %)	4(57.1)	3(42.9)	0	143±33	115±18	-	0.242*
Others (n = 27, %)	16(59.3)	10(37.0)	1(3.7)	130±33	117±29	113	0.317*
NPH (n = 30, %)	18(60.0)	11(36.7)	1(3.3)	150±33	112±15	106	<0.001*

*P* values were calculated using ANCOVA, with Bonferroni post-hoc correction to control for confounders (age, sex, and APOE ε4 genotypes).

* t-test for CC and CT.

PGRN levels are presented as mean ± standard deviation.

Abbreviations: ADD, Alzheimer’s disease dementia; CU, cognitively unimpaired people; FTD, frontotemporal dementia; MCI, mild cognitive impairment; NPH, normal pressure hydrocephalus; PD, Parkinson’s disease; PGRN, progranulin; SMI, subjective memory impairment.

### Relationship between PGRN and the rs5848 genotypes

Serum PGRN levels were not significantly different among the rs5848 genotypes in the entire sample. Even though there was a significant difference in the serum PGRN level between the CC and CT genotypes in the NPH group, no differences in the PGRN level among the rs5848 genotypes were observed in other groups (Table 3).

### Relationship between rs5848 genotypes and CSF AD biomarkers

The frequencies of the rs5848 genotypes were 31% for CT and 5% for TT in the ADD group (Table 3). Although the 40 patients with AD pathology (A+T+N+) showed a higher frequency of CT (40%) than did patients with ADD, there were no significant differences in the frequencies of the rs5848 genotypes between the patients with clinically diagnosed ADD and those with positive CSF AD biomarkers (A+T+N+) (Fisher’s exact test, p = 0.608). CSF AD biomarkers did not differ based on rs5848 genotype (Table 4). The T allele (B = -0.553, p = 0.097, odds ratio = 0.575) and the TT genotype (B = 0.097, p = 0.881, odds ratio = 1.102) had no effect on the development of AD after controlling for age, sex, and *APOE* ε4 genotype.

**Table 4:** Association between CSF biomarkers and rs5848 genotype.

N*	Aβ_1–42_ (pg/mL), median (IQR)	T-tau (pg/mL), median (IQR)	P-tau (pg/mL), median (IQR)
CC (n = 117)	598(438–898)	248(142–442)	49(34–64)
CT (n = 80)	592(427–945)	269(158–462)	56(38–72)
TT (n = 17)	799(511–940)	272(194–466)	55(42–76)
p value**	0.810	0.828	0.502

*Number of participants with available CSF data. The relationship between the rs5848 genotype and CSF p-tau levels was evaluated in the CC (n = 114), CT (n = 77), and TT (n = 17) groups, as six patients had undetectable p-tau levels.

**p values were calculated using ANCOVA, with Bonferroni post-hoc correction controlling for confounders (age, sex, diagnosis, and APOE ε4 genotypes). Log-transformed CSF biomarker levels were used in the analyses.

Abbreviations: CSF, cerebrospinal fluid; IQR, interquartile range; P-tau, phosphorylated tau; T-tau, total tau.

## Discussion

In this study, we explored serum PGRN levels, rs5848 genotypes, and their correlation with CSF AD biomarkers from individuals with neurodegenerative diseases from a Korean population. To our knowledge, this is the first study to assess serum PGRN levels based on rs5848 genotypes in an Asian population. The main finding from this study is that reduced serum PGRN levels in T allele carriers were not observed in the participants, which is in disagreement with previously published studies [15–17, 36, 37]. Although the reason for this discrepancy is not entirely clear, ethnicity associated with genetic and epigenetic factors may account for differences in the effects of the rs5848 genotypes on serum PGRN levels. A recent genome-wide association meta-analysis identified two additional genetic factors that determined PGRN concentrations: rs660240 in *CELSR2-PSRC1-MYBPHL-SORT1* and rs4747197 in *CDH23-PSAP* [38]. This meta-analysis was conducted using data from three independent European cohorts. Therefore, replicated studies in Asian populations should be conducted to identify possible ethnic differences in the effects of multiple SNPs on PGRN concentration.

Frequencies of the rs5848 genotypes did not differ between patients and controls or among patients with different neurodegenerative syndromes. The genotype frequencies in our study samples (55% for CC, 38% for CT, and 7% for TT) were comparable with those in European populations (50% for CC, 40%–42% for CT, and 8%–10% for TT) (47, 48). However, previous studies, including ours, have shown inconsistent results for specific clinical syndromes, such as FTD or ADD (12, 15, 16, 47–50). These variations may be due to differences in sample sizes or ethnicities of the study participants.

We found no significant differences in serum PGRN concentrations across clinical syndromes. Given that reduced PGRN levels have been reported in *GRN* mutation carriers [36, 39], we analyzed *GRN* mutations in all participants. *GRN* mutations are very rare in the Korean population [19, 20], and none of the participants in our study had *GRN* variants associated with pathogenesis. Instead, five heterozygous missense variants and one in-frame deletion were detected in six patients, which classified as VUS according to the guidelines of the American College of Medical Genetics and Genomics (ACMG) and Association of Molecular Pathology (AMP) and recommendation for sequence variant interpretation of ClinGen (ClinGen, https://clinicalgenome.org/working-groups/sequence-variant-interpretation/) [40]. Although the mean serum PGRN level in these six VUS carriers was not significantly different from those in controls or in patients with other neurodegenerative syndromes, one patient who had ADD and harbored c.662G >C showed serum PGRN level of 93.5 ng/mL (V4 in S1 Table and S1 Fig), which was just below the previously described cut-off value (94 ng/mL) for *GRN* mutation [11]. This variant was predicted to be deleterious by in silico algorithms (Polyphen and SIFT) and its allele frequencies of total and east Asian population from gnomAD (non-neuro cohort) were very low. The variant has been reported in sporadic progressive supranuclear palsy-like syndrome [41]. Although c.662G >C classified as VUS according to the current guidelines, a pathogenic potential of neurodegenerative disease has to be considered. In line with our study, previous studies showed that serum PGRN levels of *GRN* VUS carriers ranged from intermediate to normal [11, 42], suggesting that peripheral PGRN levels reflect the pathogenic potential (complete, partial, or no loss of function of PGRN) of the *GRN* mutation [10, 11, 42, 43].

In contrast to our observation that age, sex, MMSE score, and *APOE*ε4 genotype did not affect serum PGRN levels, several studies previously suggested that age and sex modified serum and plasma PGRN levels [17, 36, 37, 43–45]. However, one study showed results similar with those of our study [46]. Taken together, PGRN levels seem to be affected by multiple factors, including specific genetic backgrounds, epigenetic factors, epidemiologic influences, and environmental factors, in a complex manner [37].

Transgenic AD mouse models demonstrated that low brain PGRN expression enhanced Aβ_1–42_ and tau pathology [4, 5]. PGRN deficiency was also shown to reduce diffuse Aβ plaques and increased tau pathology in AD mice [6]. Based on recent evidence demonstrating the diverse roles of PGRN in the pathogenesis of AD, we investigated the relationship between CSF AD biomarkers and serum PGRN levels [47]. Despite our expectations, serum PGRN levels did not differ between patients with AD pathology (A+T+N+) and those with clinical ADD. Additionally, we found no correlation between serum PGRN levels and CSF AD biomarkers. Morenas-Rodríguez et al. however, found weak correlations between CSF PGRN and CSF AD biomarkers [46]. Although several studies have reported that plasma PGRN levels are weakly correlated with CSF PGRN levels, it is still not known whether pathophysiological changes in the central nervous system can be reliably reflected in peripheral blood biomarkers [37, 46].

Finally, we found that the T allele and the TT genotype of rs5848 did not affect the CSF AD biomarkers and did not increase risk for the development of AD. These results conflict with those from previous studies, which demonstrated that the T allele increased the CSF tau level and the TT genotype increased the risk of AD in a Taiwanese population [6, 12]. Since there have only been a few studies that examined the role of *GRN* variants in neurodegenerative diseases in Asian populations, general conclusions from each observation, including the usefulness of serum PGRN screening in the Korean population, should be made with caution until further investigations are conducted.

The main limitation of our study was the small sample size of each group. Therefore, it is possible that differences in the frequencies of rs5848 polymorphisms in clinical subgroups might not have been detected due to a lack of statistical power, rather than because of a lack of association.

In conclusion, this study, to our knowledge, is the first to assess serum PGRN levels based on rs5848 genotypes in an Asian population. We found that 1) serum PGRN levels did not significantly differ based on rs5848 genotypes; 2) there were no significant differences in rs5848 genotypes based on clinical syndromes; and 3) there were no correlations between serum PGRN levels and CSF AD biomarkers and between rs5848 genotypes and CSF AD biomarkers. We could not determine PGRN levels in *GRN* mutation carriers, as no *GRN* mutation carriers were identified in this study. Thus, we could not confirm whether serum PGRN levels were useful as a screening test for *GRN* mutation carriers in the Korean population, however, we note that our finding never means that serum PGRN screening is not effective as a biomarker for detecting *GRN* mutation. Further studies in Asian populations are required.

## Supporting information

S1 Fig Dot plot showing individual serum PGRN levels per clinical group. (PDF)

S1 Table Clinical characteristics of patients with PGRN variants of uncertain significance. (DOCX)

S1 Data (XLSX)
