# Supplementary figures and images for: Serum progranulin is not associated with rs5848 polymorphism in Korean patients with neurodegenerative diseases
Source: PLoS One. 2022 Jan 27;17(1):e0261007. doi: 10.1371/journal.pone.0261007 (PMC8794169; doi:10.1371/journal.pone.0261007)

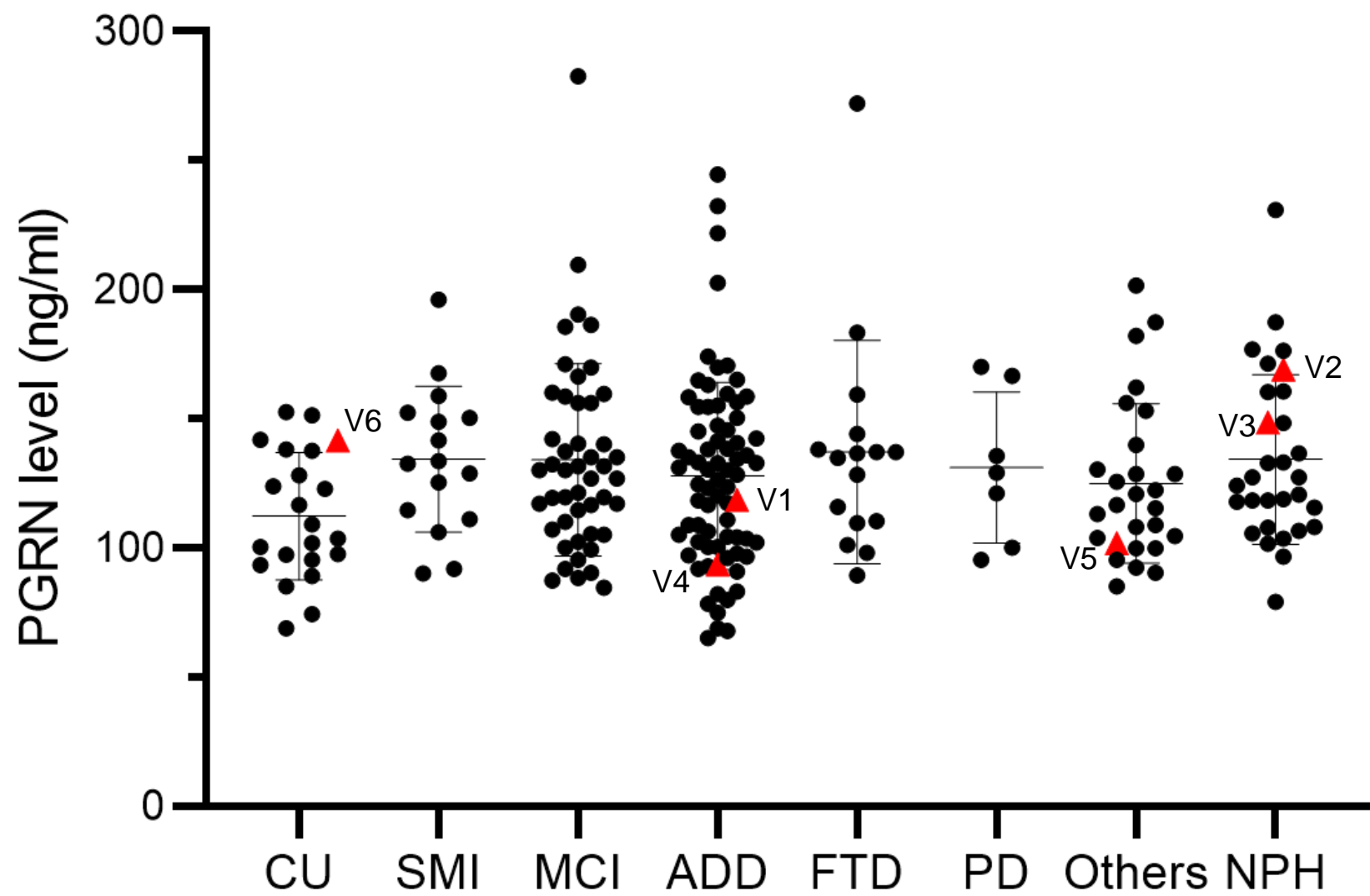

Supplement: S1 Fig — (PDF) [file pone.0261007.s001.pdf]
