# Supplementary material for: Serum progranulin is not associated with rs5848 polymorphism in Korean patients with neurodegenerative diseases
Source: PLoS One. 2022 Jan 27;17(1):e0261007. doi: 10.1371/journal.pone.0261007 (PMC8794169; doi:10.1371/journal.pone.0261007)
Supplement: S1 Table — (DOCX) [file pone.0261007.s002.docx]

S1 Table. Clinical characteristics of patients with PGRN variants of uncertain significance

| VUS ID | Variant | GRN exon | SNP ID | Allele frequency  (gnomAD,  non-neuro cohort) | | Sex | Age | Diagnosis | APOE | rs5848 genotype | Serum PGRN | CSF Amyloid | CSF T-tau | CSF P-tau | Reference |
| --- | --- | --- | --- | --- | --- | --- | --- | --- | --- | --- | --- | --- | --- | --- | --- |
|  |  |  |  | Total | East Asian |  |  |  |  |  |  |  |  |  |  |
| V1 | c.355_357del p.Asn119del hetero | Exon5 | rs758168578 | 0.00005 | 0.00067 | M | 77 | ADD | NA | CC | 118.7 | 541.9 | 472.0 | 81.4 | [1] |
| V2 | c.530G>A  p.Arg177His hetero | Exon6 | rs753441122 | 0.00002 | 0.00018 | F | 78 | NPH | ε3/ε3 | CC | 169.0 | 223.6 | 153.5 | 27.5 |  |
| V3 | c.530G>A  p.Arg177His hetero | Exon6 | rs753441122 | 0.00002 | 0.00018 | F | 66 | NPH | NA | CC | 148.7 | 779.3 | 109.4 | 27.9 |  |
| V4 | c.662G>C  p.Cys221Ser hetero | Exon7 | rs758322775 | 0.00009 | 0.00120 | F | 77 | ADD | ε3/ε4 | CT | 93.5 | 595.0 | 447.8 | 69.9 | [1] |
| V5 | c.827C>T  p.Ala276Val hetero | Exon8 | rs202178902 | 0.00005 | 0 | M | 65 | Unspecified dementia | ε2/ε4 | CC | 101.8 | 864.0 | 236.4 | 54.5 | [2], [3] |
| V6 | c.1690C>T  p.Arg564Cys hetero | Exon13 | rs200419979 | 0.00001 | 0.00015 | M | 74 | CU | NA | CC | 141.6 | 1175.0 | 280.0 | 50.0 | [4] |

Segregation data in affected family members is impossible because no one had family history.

Allele frequency is from The Genome Aggregation Database (gnomAD).

Nucleotides are numbered according to the reference cDNA sequence, GenBank accession number NM_002087.4.

Abbreviations: ADD, Alzheimer’s disease dementia; APOE, apolipoprotein E; CSF, cerebrospinal fluid; CU, cognitively unimpaired people; F, female;GRN, granulin; M, male; NA, not available; NPH, normal pressure hydrocephalus; PGRN, progranulin; P-tau, phosphorylated tau; SNP, single nucleotide polymorphism; T-tau, total tau

Reference

1. Yabe, I., et al., *Mutations in bassoon in individuals with familial and sporadic progressive supranuclear palsy-like syndrome.* Sci Rep, 2018. **8**(1): p. 819.

2. Orme, T., et al., *Analysis of neurodegenerative disease-causing genes in dementia with Lewy bodies.* Acta Neuropathol Commun, 2020. **8**(1): p. 5.

3. Yu, C.E., et al., *The spectrum of mutations in progranulin: a collaborative study screening 545 cases of neurodegeneration.* Arch Neurol, 2010. **67**(2): p. 161-70.

4. Sleegers, K., et al., *Serum biomarker for progranulin-associated frontotemporal lobar degeneration.* Ann Neurol, 2009. **65**(5): p. 603-9.
